# Effect Evaluation of Dexmedetomidine Intravenous Anesthesia on Postoperative Agitation in Patients with Craniocerebral Injury by Magnetic Resonance Imaging Based on Sparse Reconstruction Algorithm

**DOI:** 10.1155/2022/5161703

**Published:** 2022-06-23

**Authors:** Xue Feng, Binbin Zhao, Yongqiang Wang

**Affiliations:** ^1^Department of Anesthesia Surgery, First Affiliated Hospital, Heilongjiang University of Traditional Chinese Medicine, Harbin 150040, Heilongjiang, China; ^2^Department of Outpatient, Harbin Red Cross Central Hospital, Harbin 150076, Heilongjiang, China

## Abstract

The effect of dexmedetomidine on postoperative agitation of patients with craniocerebral injury was investigated based on magnetic resonance imaging (MRI) with the sparse reconstruction algorithm. Sixty patients with craniocerebral injury who underwent tracheal intubation and craniotomy hematoma removal under general anesthesia in hospital were selected as the research objects. Patients were randomly and averagely divided into the normal saline group (group A) and the dexmedetomidine (DEX) group (group B). DEX was added to patients in group A during anesthesia. Other operations in group B were the same as those in group A, where DEX needed to be used was replaced by an equal amount of the normal saline. All patients received the MRI examination, and the images were processed by using the sparse reconstruction algorithm. After the surgery, some indexes, such as hemodynamics (mean arterial pressure (MAP) and hear rate (HR)), the Riker sedation agitation score, the Ramsay sedation score, and the visual analogue scale (VAS) score were recorded and compared. The results showed that the MRI image quality processed by sparse reconstruction algorithm was observably improved. After reconstruction, the sharpness of the image was significantly improved, and the distinction between lesions and tissues was also increased. The Riker sedation agitation score and the incidence of agitation in group A were greatly lower than those in group B (16% VS 76%, *P* < 0.05). The Ramsay sedation score of group A was manifestly higher than that of group B. The cases of postoperative nausea, vomiting, chills, delirium, and bradycardia in group A were 2, 1, 1, 0, and 1, respectively. The cases of postoperative nausea, vomiting, chills, delirium, and bradycardia in group B were 3, 9, 6, 5, and 0, respectively. The cases of chills and delirium in group A were observably less than those in group B (*P* < 0.05). In conclusion, based on the sparse reconstruction algorithm, the MRI technology and DEX had high adoption value in preventing postoperative agitation of patients with craniocerebral injury. Compared with group B, the hemodynamics of patients in group A was more stable.

## 1. Introduction

In recent years, the incidence of craniocerebral injury is increasing gradually. At present, it has become a relatively common clinical acute trauma, and it is also one of the most common causes of patients' death in both the emergency department and the neurosurgery department [1]. Currently, the main method for clinical treatment for craniocerebral injury is the craniotomy evacuation of the hematoma. The craniotomy evacuation of hematoma is generally performed under tracheal intubation and general anesthesia, which can not only cause hemodynamic fluctuations but also lead to a linear increase in the incidence of agitation in patients during awakening [2]. A large number of clinical data show that patients with craniocerebral injury often have complications, such as the fluctuation of heart rate and blood pressure and the dysphoria after craniotomy. In some serious cases, patients even have secondary cerebral ischemia and hypoxia, which further aggravates brain tissue injury [3]. Hence, the appropriate sedation therapy for patients with craniocerebral injury can reduce various stress reactions caused by tracheal stimulation. The treatment of sedation and analgesia has become an essential treatment for patients with craniocerebral injury [4]. Nevertheless, the sedative and analgesic drugs generally cause respiratory depression, effect on consciousness, and pupil light reflex, which usually bring great interference to the doctor's judgment [5]. Dexmedetomidine (DEX) produces dose-dependent sedation that is similar to the status of natural sleep. Simultaneously, it also has abirritation, diuretic effect, and anti-sympathetic activity as well as a protective effect on the heart, brain, and kidney [6]. DEX has been widely used in clinical treatment, and its efficacy has been recognized by the majority of doctors. However, there are few clinical investigations on the effect of DEX on postoperative agitation in patients with craniocerebral injury, which needs to be further explored.

Computed tomography (CT) is a commonly used imaging method for the diagnosis of the craniocerebral injury. CT provides great help for the diagnosis of craniocerebral injury, and it has many advantages, such as low cost and scanning time. However, the CT scan cannot detect the skull base, posterior fossa, and non-hemorrhagic injuries [7]. Due to its inability to examine shear injuries of white matter, corpus callosum, and brainstem, the CT scan has little help in the examination of the patients with severe illness and does not show substantial advantages in the long-term prognosis [8]. With the continuous development and progress of imaging technology, magnetic resonance imaging (MRI) has been rapidly developed, which provides the reference and basis for the diagnosis of craniocerebral injury. According to domestic and overseas investigations, MRI can reveal more detailed lesions than CT. Besides, hemorrhagic and non-hemorrhagic injuries can also be clearly distinguished. Zhao et al. (2019) studied the diagnostic effect of MRI on patients with craniocerebral injury, and the results showed that the accuracy of MRI in diagnosing craniocerebral injury could reach 93% [9]. MRI has high specificity and sensitivity in the diagnosis of cortical, brainstem, and cerebellum injuries [10].

MRI technology has been widely applied in modern clinical medicine. Nonetheless, the long imaging time of MRI has always been a vital problem that restricts its further development. The average imaging time of the latest third-generation cone-beam CT is within a few seconds, that of the spiral CT is even faster at about 1 second, and that of the conventional MRI is at about 15 to 30 seconds [11]. The main factors that affect MRI imaging are analyzed, which are mainly classified into the machine-scanning time and the image reconstruction time. Recently, computer technology has developed rapidly, and it has achieved good results in the MRI image reconstruction [12]. Currently, the unpaid image reconstruction time has been reduced to the order of milliseconds [13]. The artificial intelligence (AI) algorithm was applied for the processing of MRI images and evaluation of patients with craniocerebral injury under DEX intravenous anesthesia. The effect of DEX on patients' postoperative agitation was analyzed. This was of great significance for the promotion and adoption of the AI algorithm in the medical field and for the reduction of the incidence of postoperative agitation and other adverse reactions in patients under general anesthesia.

## 2. Methods

### 2.1. Research Objects

Sixty patients with craniocerebral injury who underwent tracheal intubation and craniotomy hematoma removal under general anesthesia in hospital from January 2020 to March 2021 were selected as the research objects. There were 36 male patients and 24 female patients, with a mean age of 44.7 ± 11.3 years old. Patients were randomly and averagely divided into the normal saline group (group A) and the dexmedetomidine (DEX) group (group B). All the patients signed the informed consent, and the experiment satisfied the requirements of medical ethics.

The inclusion criteria were as follows. I. Patients diagnosed with craniocerebral injury by CT and MRI; II. Patients with a Glass score (GCS) of 9 to 12 points at admission; III. Patients with admission time of 1 to 6 hours; IV. Patients who were no younger than 18 years old. The exclusion criteria were as follows. I. Patients who needed a second surgery; II. Patients with other severe injuries; III. Patients with insufficiency of liver, kidney, lung, and heart; IV. Patients who took a long-term use of psychotropic drugs; V. Patients with severe allergies; VI. Patients with drug addiction or alcoholism; VII. Patients with physical disabilities; VIII. Those with mental disorders or illnesses that cannot communicate with the doctor.

### 2.2. Anesthesia Methods

All patients received intramuscular injections of 0.5 mg and atropine and 0.1 g phenobarbital 30 minutes before surgery. After patients entered the operating room, they received oxygen through a mask with an oxygen flow rate of 3 L/min. Routine indexes were detected, such as heart rate, non-invasive blood pressure, pulse oxygen saturation, and electrocardiogram. After patients calmed down, their vital signs were measured three times, and the average value was calculated as the basic value. The peripheral venous channel was established for two weeks, and the compound sodium chloride injection was given. Continuous arterial pressure was monitored and recorded through the routine arteria dorsalis pedis catheterization. At the beginning of anesthesia induction, patients in group A were intravenously pumped with Dex1*μ*g/kg for no less than 10 minutes. Patients in group B were given the same volume of normal saline intravenously at the same speed and time.

For anesthesia induction, midazolam 0.1 mg/kg and sufentanil 0.5 *μ*g/kg were injected intravenously successively. After patients lost consciousness, they were injected with cisatracurium besilate 0.2 mg/kg and propofol (Di ShiNing) 2 mg/kg. After 3 minutes of pressurized nitrogen removal and oxygen delivery, the orotracheal intubation was performed under direct vision. After confirmation, ventilation was controlled by connecting the A5 anesthesia machine. Tidal volume was set at 8 ml/kg, oxygen flow was set at 1.5 L/min, respiratory rate was set at 12 times/min, and inhalation/respiration ratio was 1 : 2.

For the maintenance of anesthesia, the maintenance amount of DEX0.5 *μ*g/kg.h was intravenously pumped in group A, and the same volume of normal saline was pumped at the same speed in group B. Both groups received the continuous intravenous pumping of propofol 4–12 mg/kg.h, cisatracurium besilate 1.5 *μ*g/kg.min, and remifentanil 0.05-2ug/kg·min to maintain anesthesia. Compound sodium chloride injection and hydroxyethyl starch 130/0.4 sodium chloride injection were used. During the surgery, respiratory parameters were adjusted according to the results of arterial blood gas detection, and the partial pressure of end-tidal pressure of carbon dioxide (PETC02) was maintained at 30–40 mmHg. The infusion speed of anesthetics was adjusted according to the arterial blood pressure, and the fluctuation of arterial pressure was controlled within 30% of the base value. The infusion of muscle relaxant and DEX was stopped simultaneously when the galea aponeurotica was sutured in the surgery.

After the surgery, all anesthetic drugs were stopped. The tracheal tube was removed after patients became conscious and reached extubation indexes.

### 2.3. MRI Examination

1.5 T MRI equipment was adopted to perform a general scan of the head. The detailed scanning parameters were as follows. The T2 weighted imaging (T2WI)/fast spin echo (FSE), T1 weighted imaging (T1WI)/IR transverse axis, and T1WI/IR sagittal. For T2WI, the time of repetition (TR) was 4000 ∼ 4500 ms and the time of echo (TE) was 100 ms; for T1WI, it was 1750 ms. The layer thickness was 6 ∼ 8 mm, and the layer interval was 0.5 ∼ 1.0 mm. Nex was 1 ∼ 2 times, the matrix was 384 × 256, and the field of view (FoV) was 24 cm.

All patients underwent axial, sagittal, and coronal scans. MRI images were evaluated by three senior radiologists who had no prior knowledge of patients' injury history or disease. If there was any dispute about the three-dimensional judgment, the three radiologists needed to discuss and make the conclusion. The number, location, and signal intensity of the injury were recorded. Scans were generally performed 1–39 days after injury. The scanning time ranged from about 17 to 35 minutes. Sedation and endotracheal intubation were required in some patients during the examination. Blood pressure and high concentration of peripheral blood pressure required continuous monitoring.

### 2.4. MRI Image Processing Based on Sparse Reconstruction Algorithm

A sparse reconstruction algorithm was proposed according to the characteristics of the back-projection algorithm. Firstly, the algorithm was optimized. Then, in the following equation, according to the properties of trigonometric functions, sines and cosines of angles that differed by 90 degrees were converted.

(1) $$\begin{array}{c} sin\left(\alpha +90\right)=cos\left(\alpha \right)cos\left(\alpha +90\right)=-sin\left(\alpha \right),\\ sin\left(\alpha +180\right)=-\sin\left(\alpha \right)cos\left(\alpha +180\right)=-\cos\left(\alpha \right),\\ \sin\left(\alpha +270\right)=-cos\left(\alpha \right)\cos\left(\alpha +270\right)=sin\left(\alpha \right). \end{array}$$

According to the following equations, sine and cosine operations of (*β* − *ϕ*) were simplified.

(2) $$\begin{array}{c} U\left(r,\phi ,\beta \right)=\frac{D+r\;\sin\left(\beta -\phi \right)}{D}, \end{array}$$

(3) $$\begin{array}{c} s{}^{\prime }=D\frac{r\;\cos\left(\beta -\phi \right)}{D+r\;\sin\left(\beta -\phi \right)}. \end{array}$$

In equations (2) and (3), the values of *β* and *ϕ* ranged from 0 to 360 degrees. The reconstruction area was divided into four quadrants, and the projection data was also divided into four regions according to the projection angle, namely, 0 < *β*_1_ ≤ 90,90 < *β*_2_ ≤ 180,180 < *β*_3_ ≤ 270,270 < *β*_4_ ≤ 360. Four spots were selected during the reconstruction, namely, *E*(*r*, *ϕ*), *E*_1_(*r*, *ϕ*+90), *E*_2_(*r*, *ϕ*+180), *E*_3_(*r*, *ϕ*+270). These four spots belonged to the four quadrants, and the values of *r* were equal. Besides, their *ϕ* values differed by 90 degrees in turn.

Reconstruction steps after remodeling were as follows.

Firstly, for the spot *E*(*r*, *ϕ*) in the first quadrant, when 0 < *β*_1_ ≤ 90, *r* sin(*β*_1_ − *ϕ*) and *r* cos(*β*_1_ − *ϕ*) were calculated. Then, according to equations (2) and (3), *U* and *s*′ were calculated as shown in the following equations.

(4) $$\begin{array}{c} U\left(r,\phi ,\beta_{1}\right)=\frac{D+r\;\sin\left(\beta_{1}-\phi \right)}{D}, \end{array}$$

(5) $$\begin{array}{c} s^{\prime }\left(r,\phi ,\beta_{1}\right)=D\frac{r\;\cos\left(\beta_{1}-\phi \right)}{D+r\;\sin\left(\beta_{1}-\phi \right)}. \end{array}$$

Secondly, in the following equation, for the spot *E*1(*r*, *ϕ*+90), *β*_2_=*β*_1_+90 was set, and the values of *r* were set to be equal.

(6) $$\begin{array}{c} \beta_{2}-\left(\phi +90\right)=\beta_{1}+90-\phi -90=\beta_{1}-\phi . \end{array}$$

Hence, for the spot *E*(*r*, *ϕ*) in the first quadrant, there was only one corresponding spot *E*1(*r*, *ϕ*+90) in the second quadrant. The *U* and *s*′ of the two spots corresponded to each other. For *β*_2_=*β*_1_+180, *U* and *s*′ of *E*2(*r*, *ϕ*+180) were equal to those of *E*(*r*, *ϕ*). For *β*_4_=*β*_1_+270, *U* and *s*′ of *E*3(*r*, *ϕ*+270) were equal to those of *E*(*r*, *ϕ*). The following equations showed the calculation methods.

(7) $$\begin{array}{c} U\left(r,\phi ,\beta_{1}\right)=U\left(r,\phi +90,\beta_{2}\right)\\ =U\left(r,\phi +180,\beta_{3}\right)\\ =U\left(r,\phi +270,\beta_{4}\right). \end{array}$$

(8) $$\begin{array}{c} s^{\prime }\left(r,\phi ,\beta_{1}\right)=s^{\prime }\left(r,\phi +90,\beta_{2}\right)\\ =s^{\prime }\left(r,\phi +270,\beta_{4}\right). \end{array}$$

In this step, the values of *U* and *s*′ of the four spots were calculated once only, which helped save the time.

Thirdly, in the following equations, when 0 < *β*_1_ ≤ 90, the values of *U* and *s*′ of *E*1(*r*, *ϕ*+90) in the second quadrant were calculated.

(9) $$\begin{array}{c} U\left(r,\phi +90,\beta_{1}\right)=\frac{D+r\;\sin\left(\beta_{1}-\phi -90\right)}{D}\\ =\frac{D-r\;\cos\left(\beta_{1}-\phi \right)}{D}, \end{array}$$

(10) $$\begin{array}{c} s^{\prime }\left(r,\phi +90,\beta_{1}\right)=D\frac{r\;\cos\left(\beta_{1}-\phi -90\right)}{D+r\;\sin\left(\beta_{1}-\phi -90\right)}\\ =D\frac{r\;\sin\left(\beta_{1}-\phi \right)}{D-r\;\cos\left(\beta_{1}-\phi \right)}. \end{array}$$

*r* sin(*β*_1_ − *ϕ*) and *r* cos(*β*_1_ − *ϕ*) were both obtained in the first step, so a floating point operation was performed in this step. Then, the following equations were obtained.

(11) $$\begin{array}{c} U\left(r,\phi +90,\beta_{1}\right)=U\left(r,\phi +180,\beta_{2}\right)\\ =U\left(r,\phi +270,\beta_{3}\right)\\ =U\left(r,\phi ,\beta_{4}\right). \end{array}$$

(12) $$\begin{array}{c} s^{\prime }\left(r,\phi +90,\beta_{1}\right)=s{}^{\prime }\left(r,\phi +180,\beta_{2}\right)\\ =s{}^{\prime }\left(r,\phi +270,\beta_{3}\right)\\ =s{}^{\prime }\left(r,\phi ,\beta_{4}\right). \end{array}$$

The values of *U* and *s*′ for spots *E*2(*r*, *ϕ*+180) and *E*3(*r*, *ϕ*+270) could be calculated in the similar way.

After image reconstruction, the mean square error (MSE), peak signal-to-noise ratio (PSNR), structural similarity (SSIM), and other indicators were used to quantitatively evaluate the image reconstruction effect. The specific calculation methods of the three indicators were as follows:

(13) $$\begin{array}{c} \text{MSE}=\frac{1}{mn}{\sum_{i=0}^{m-1}\sum_{j=0}^{n-1}\left[I\left(i,j\right)-K\left(i\cdot j\right)\right]}^{2}, \end{array}$$

(14) $$\begin{array}{c} PSNR=10\cdot \;\log_{10}\left(\frac{{\text{MAX}}_{I}^{2}}{\text{MSE}}\right)=20\cdot \;\log_{10}\left(\frac{{\text{MAX}}_{I}}{\sqrt{\text{MSE}}}\right), \end{array}$$

(15) $$\begin{array}{c} SSIM\left(x,y\right)=\frac{\left(2\mu_{x}\mu_{y}+c_{1}\right)\left(2\sigma_{\mathrm{xy}}+c_{2}\right)}{\left(\mu_{x}^{2}\right)+\mu_{y}^{2}+c_{1}\left(\sigma_{x}^{2}+\sigma_{y}^{2}+c_{2}\right)}. \end{array}$$

### 2.5. Observation Index

Firstly, respiratory recovery time, wake-up time, and extubation time were recorded. Secondly, heart rate (HR) (times/min) and mean arterial pressure (MAP) (mmHg) were recorded immediately after awakening (T1), immediately after extubation (T2), 5 minutes (T3), 30 minutes (T4), 60 minutes (T5), and 120 minutes (T6) after extubation in the two groups. Thirdly, the Riker sedation agitation scores of patients in the two groups at 6 postoperative time points were recorded [14]. Fourthly, the degree and incidence of agitation were recorded from the end of surgery to 120 minutes after extubation. Fifthly, the Ramsay sedation score of the patients in the two groups at 6 postoperative points was recorded [15]. Sixthly, the visual analogue scale (VAS) scores of patients in the two groups at 6 postoperative points were recorded. Seventhly, the total amount of remifentanil and propofol used in the two groups was recorded, and the average dose used in each group was calculated. Finally, the incidence of adverse reactions from the end of surgery to 120 minutes after extubation in the two groups was recorded.

### 2.6. Statistical Analysis

From thisSPSS 22.0 was used for data statistics and analysis. Mean ± standard deviation (*x* ± *s*) was how measurement data were expressed. Comparison between the two groups was performed by *t* test. Analysis of variance was used for the comparisons within the groups. Enumeration data were tested by *χ*^2^ test. The difference was statistically considerable with *P* < 0.05.

## 3. Results

### 3.1. Patients' Classic Images

**Figure 1:**
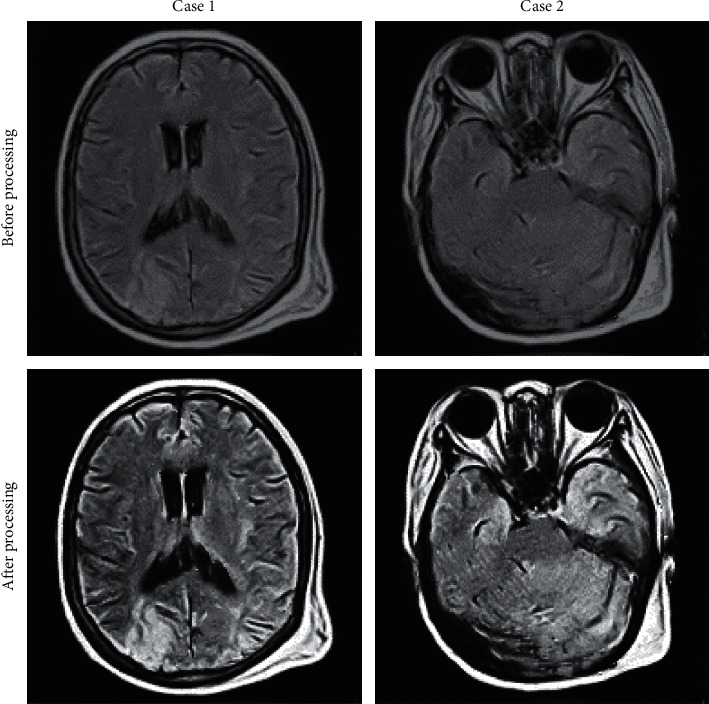
Presentation of images of typical cases.

Figure 1 shows the images of typical cases. The MRI images processed by the sparse reconstruction algorithm had higher sharpness and more prominent details on the edges of lesions compared with the unprocessed MRI images, which indicated that the image quality was obviously improved.

### 3.2. Quantitative Evaluation of Algorithm Image Reconstruction Effect

**Figure 2:**
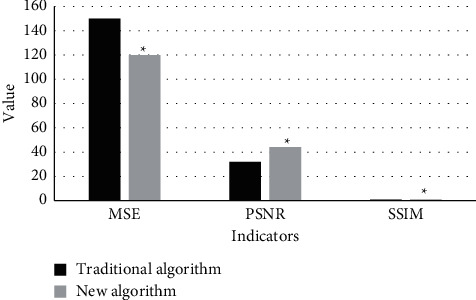
Quantitative evaluation of algorithm image reconstruction effect. ^*∗*^Compared with the traditional algorithm, *P* < 0.05.

Figure 2 shows the quantitative evaluation results of the image reconstruction effect of the traditional algorithm and the new algorithm proposed in this work. Analysis of Figure 2 showed that the MSE, PSNR, and SSIM of the traditional algorithm were 150, 32, and 0.77, respectively; while those of the new algorithm were 120, 44, and 0.92, respectively. It can be known that there was a significant difference in the indicators of the two algorithms (*P* < 0.05). This suggested that the performance of the new algorithm proposed in this work was significantly better than the traditional algorithm in the reconstruction of MRI images of patients with craniocerebral injury.

### 3.3. Comparison of the General Recovery Time

**Figure 3:**
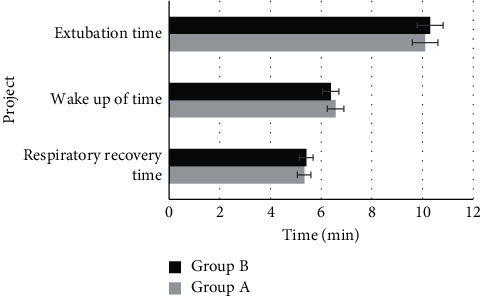
Comparison of the general recovery time between the two groups.

Figure 3 shows the statistical results of postoperative respiratory recovery time, wake-up time, and extubation time of patients in the two groups. The respiratory recovery time, wake-up time, and extubation time in group A were 5.33 ± 1.3, 6.57 ± 2.4, and 10.1 ± 3.3, respectively. The respiratory recovery time, wake-up time, and extubation time in group B were 5.41 ± 2.2, 6.38 ± 1.4, and 10.3 ± 2.7, respectively. There was insignificant difference in the general recovery time between the two groups (*P* > 0.05).

### 3.4. Comparison of Hemodynamic Data

**Figure 4:**
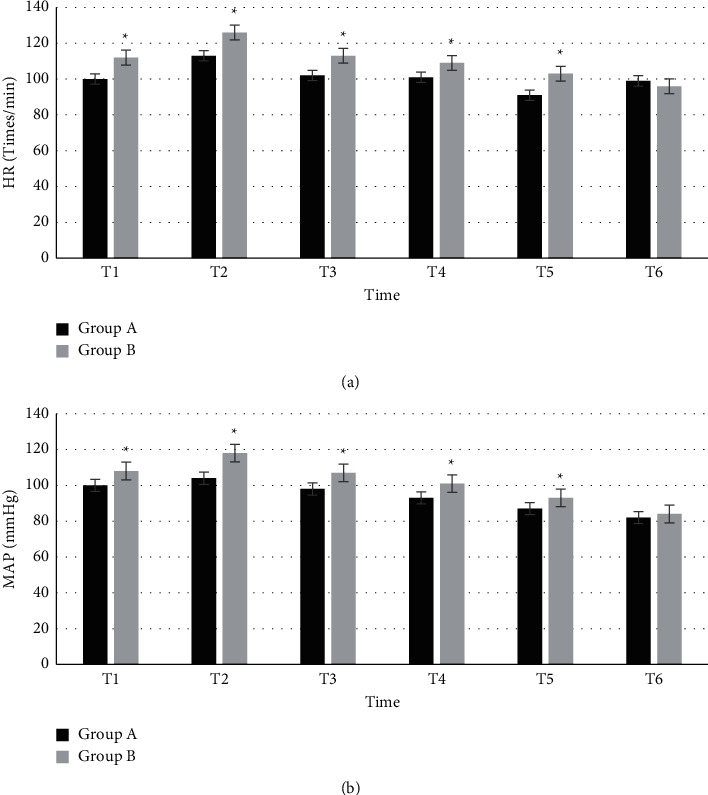
Comparison of hemodynamic data between the two groups at each time point. ^*∗*^Compared with group A, *P* < 0.05.

Figure 4 shows the comparison of hemodynamics between the two groups at each time point. HR of group A at T1, T2, T3, T4, T5, and T6 were 100 ± 5.5, 113 ± 6.8, 102 ± 7.7, 101 ± 7.1, 91 ± 6.6, and 99 ± 7.3, respectively. MAP of group A at T1, T2, T3, T4, T5, and T6 were 100 ± 5.7, 104 ± 6.1, 98 ± 8.1, 93 ± 9.2, 87 ± 6.6, and 82 ± 7.7, respectively. In group B, HR at T1, T2, T3, T4, T5, and T6 were 112 ± 6.8, 126 ± 7.4, 113 ± 4.9, 109 ± 6.3, 103 ± 8.8, and 96 ± 9.1, respectively. MAP of group B at T1, T2, T3, T4, T5, and T6 were 108 ± 7.8, 118 ± 8.8, 107 ± 6.9, 101 ± 7.6, 93 ± 6.3, and 84 ± 7.2, respectively. The comparison between the two groups showed that there were significant differences in HR and MAP at each time point within 60 minutes (*P* < 0.05).

### 3.5. Comparison of the Riker Sedation Agitation Scores after the Surgery

**Figure 5:**
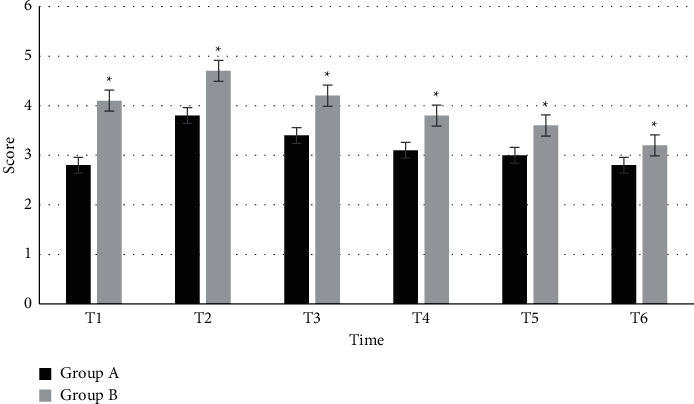
Comparison of the Riker sedation agitation scores at each time point between the two groups. ^*∗*^Compared with group A, *P* < 0.05.

Figure 5 shows the comparison of the Riker sedation agitation scores of patients in the two groups after the surgery. In group A, the Riker sedation agitation scores at T1, T2, T3, T4, T5, and T6 were 2.8 ± 0.3, 3.8 ± 0.1, 3.4 ± 0.4, 3.1 ± 0.3, 3 ± 0.2, and 2.8 ± 0.5, respectively. In group B, the Riker sedation agitation scores at T1, T2, T3, T4, T5, and T6 were 4.1 ± 0.3, 4.7 ± 0.4, 4.2 ± 0.2, 3.8 ± 0.3, 3.6 ± 0.22, and 3.22 ± 0.31, respectively. The difference was statistically considerable in the Riker sedation agitation score within 60 minutes between the two groups (*P* < 0.05).

### 3.6. Comparison of the Incidence of Agitation

Table 1 shows the comparison of the incidence of agitation between the two groups. The incidence of agitation was 16% in group A and that was 76% in group B. The incidence of agitation in group A was remarkably lower than that in group B (*P* < 0.05).

### 3.7. The Ramsay Sedation Score

**Figure 6:**
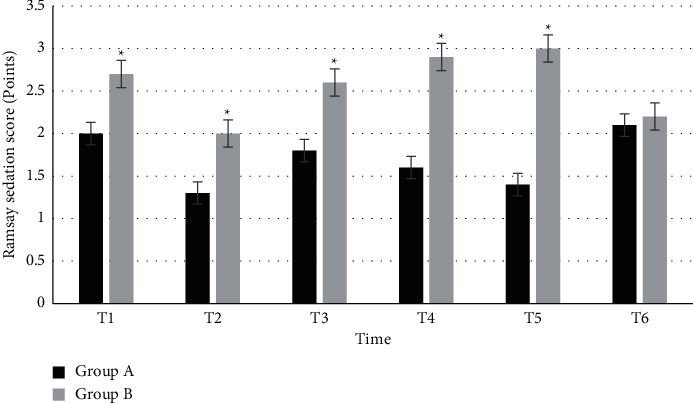
Comparison of the Ramsay sedation scores between the two groups at each time. ^*∗*^Compared with group A, *P* < 0.05.

Figure 6 shows the comparison of the Ramsay sedation scores between the two groups at each time. The Ramsay sedation scores at T1, T2, T3, T4, T5, and T6 in group A were 2 ± 0.1, 1.3 ± 0.3, 1.8 ± 0.2, 1.6 ± 0.5, 1.4 ± 0.7, and 2.1 ± 0.4, respectively. In group B, the Ramsay sedation scores at T1, T2, T3, T4, T5, and T6 were 2.7 ± 0.4, 2 ± 0.3, 2.6 ± 0.2, 2.9 ± 0.1, 3 ± 0.3, and 2.2 ± 0.4, respectively. There was a statistically significant difference in the Ramsay sedation scores within 60 minutes between the two groups (*P* < 0.05).

### 3.8. Comparison of the VAS Scores at Each Time Point

**Figure 7:**
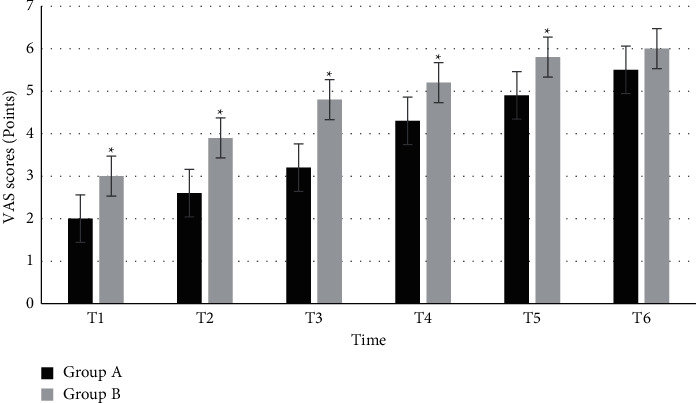
Comparison of the VAS scores at each time point between the two groups. ^*∗*^Compared with group A, *P* < 0.05.

Figure 7 shows the comparison of the VAS scores at each time point between the two groups. The VAS scores of group A at T1, T2, T3, T4, T5, and T6 were 2 ± 0.2, 2.6 ± 0.3, 3.2 ± 0.5, 4.3 ± 0.4, 4.9 ± 0.2, and 5.5 ± 0.3, respectively. The VAS scores of group B at T1, T2, T3, T4, T5, and T6 were 3 ± 0.2, 3.9 ± 0.4, 4.8 ± 0.6, 5.2 ± 0.3, 5.8 ± 0.2, and 6 ± 0.5, respectively. Within 60 minutes, the VAS scores of group A were evidently higher than those of group B at each time point (*P* < 0.05).

### 3.9. Comparison of Dosages of Remifentanil and Propofol

**Figure 8:**
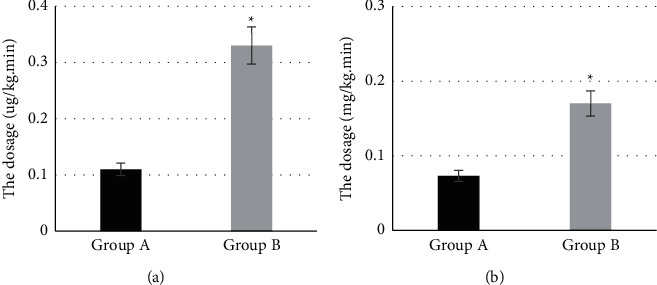
Comparison of dosages of remifentanil and propofol between two groups. ^*∗*^Compared with group A, *P* < 0.05.

Figure 8 shows the comparison of dosages of remifentanil and propofol between two groups. The average dosage of remifentanil and propofol in group A were 0.11 ± 0.01 and 0.073 ± 0.03, respectively. The average dosage of remifentanil and propofol in group B were 0.33 ± 0.02 and 0.17 ± 0.015, respectively. The average dosage of remifentanil and propofol in group A were markedly lower than those in group B (*P* < 0.05).

### 3.10. Comparison of Postoperative Adverse Reactions between the Two Groups

**Figure 9:**
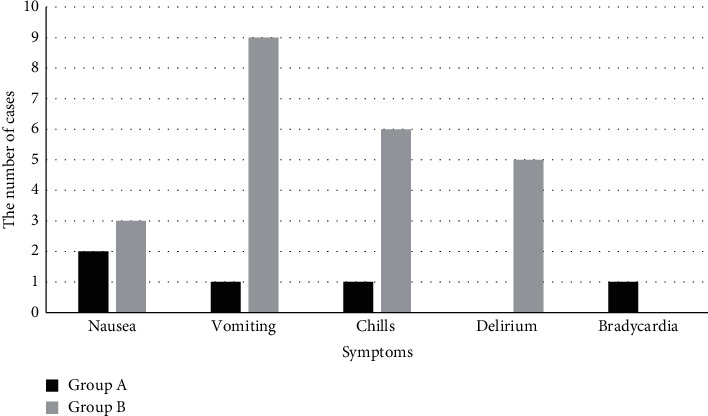
The conditions of postoperative adverse reactions in the two groups.

Figure 9 shows the comparison of postoperative adverse reactions between the two groups. The cases of postoperative nausea, vomiting, chills, delirium, and bradycardia in group A were 2, 1, 1, 0, and 1, respectively. The cases of postoperative nausea, vomiting, chills, delirium, and bradycardia in group B were 3, 9, 6, 5, and 0, respectively. The cases of chills and delirium in group A were observably less than those in group B (*P* < 0.05).

## 4. Discussion

Postoperative agitation is defined as the over-excitability of patients during the waking period under anesthesia with ether, cyclopropane, and ketamine. Its main clinical manifestations are unconscious movements of the body, uncontrollable crying, irrational language, and excited agitation. Agitation occurs for several reasons, such as surgically related factors, anesthetic factors, and adverse stimuli. At present, the mechanism of postoperative agitation cannot be precisely explained [16, 17]. Some scholars believe that the occurrence of postoperative agitation is related to the different degrees of inhibition of the central nervous system by anesthetic drugs [11, 18]. Postoperative agitation is a common but difficult complication to be controlled. Postoperative agitation is dangerous in patients with craniocerebral injury. It can not only interfere with the observation of postoperative conditions but also seriously affect the respiratory and circulatory functions of patients and further lead to a substantial increase in intracranial pressure [19, 20]. The probability of intracranial hemorrhage also increases, and postoperative agitation also induces secondary brain injury like the aggravation of cerebral edema. Moreover, patients have such problems as disturbance of consciousness, which leads to the occurrence of accidents during the removal of the tracheal tube, urinary tube, and drainage tube. This will not only bring safety threats to patients but also increase the difficulty of postoperative nursing [21].

Sedative and analgesic therapy can reduce restlessness and stress response, which plays a crucial role in improving the prognosis of patients with craniocerebral injury [22]. Benzodiazepines, propofol, and opioids are often used in the clinic to reduce the occurrence of agitation. However, these drugs have such side effects as respiratory depression and urinary retention. These side effects have a great interference effect on the clinical observation of patients' conditions, so its clinical adoption is limited to a certain extent. DEX is a kind of *α*2 adrenergic receptor agonist that is discovered and applied late in the clinic. It has a unique effect of calming but not inhibiting respiration [23], so it is widely used in the clinic. The craniotomy evacuation of the hematoma is a very common and vital treatment for patients with craniocerebral injury. Effective sedative and analgesic therapy after the surgery can effectively prevent agitation in patients who have undergone neurosurgical operations [24]. DEX can reduce the excitability of the sympathetic nervous system and reduce the hemodynamic changes caused by the stress response, which plays the role of analgesia and sedation. Consequently, it is applied in the anesthesia and surgery of patients with craniocerebral injury. Patients with traumatic brain injury were selected as the research subjects in this work to observe the effect of dexmedetomidine on postoperative agitation. The results showed that compared with group B, patients in group A were hemodynamically more stable. The scores of Rick sedation and the incidence of agitation in group A were significantly lower than those in group B. The Ramsay sedation score in group A was significantly higher than that in group B, and the incidence of postoperative complications in group A was also significantly lower than that in group B. Therefore, DEX has high clinical application value in reducing postoperative agitation in patients with craniocerebral injury.

Accurate diagnosis of the craniocerebral injury is of great significance to the treatment of the disease. Only by an accurate judgment of the degree of injury can correct treatment measures be taken. Imaging techniques play an important role in the diagnosis of these diseases, among which CT and MRI are more commonly used and more concerned. According to many investigations, MRI is more sensitive than CT in the diagnosis of craniocerebral injury, and MRI also has great advantages in the evaluation of the prognosis of craniocerebral injury [25]. In recent years, with the rapid development of computer technology, the combination of computer technology and other technologies becomes the main trend of the development of various fields. In the medical field, all kinds of medical image processing technology are explored, and there is quite good progress in computer technology applied in the field of medical image processing [26]. In the image processing of MRI, the sparse reconstruction algorithm is of great concern [27]. The MRI images of all patients were processed by sparse reconstruction algorithm, and the value of this algorithm in processing MRI images was studied. The results showed that the quality of MRI images processed by the sparse reconstruction algorithm was significantly improved. Compared with the traditional algorithm, the sparse reconstruction algorithm had better performance in image processing. This shows that the MRI based on sparse reconstruction algorithm also has good performance in the diagnosis of traumatic brain injury.

## 5. Conclusion

Patients with craniocerebral injury were selected as research objects. The effect of DEX on postoperative agitation was observed. The sparse reconstruction algorithm was used to process MRI images of all patients, and the value of the algorithm in processing MRI images was explored. The results reflected that the quality of MRI images processed by the sparse reconstruction algorithm was evidently improved. Compared with group B, the Riker sedation agitation score, the Ramsay sedation score, hemodynamics, and other indexes of group A were better. In conclusion, DEX had a high clinical adoption value in reducing postoperative agitation in patients with craniocerebral injury. Besides, MRI based on sparse reconstruction algorithm had good performance in the diagnosis of the craniocerebral injury. There were still some limitations in this work. For example, it only studied the image reconstruction performance of two algorithms, and many excellent algorithms had not been introduced. Therefore, the algorithm proposed in this work was not an optimal processing algorithm. In addition, it only analyzed and showed the results of MRI examinations and failed to compare with other examination methods, which may lead to certain errors in the research results. In the future study and work, it would study and improve the above problems, and continue such research in a comprehensive and in-depth manner.

## Figures and Tables

**Table 1:** Comparison of the incidence of agitation between the two groups.

Grading	Group A	Group B
3	5	2
4	20	5
5	3	7
6	2	9
7	0	7
Incidence of agitation (%)	16	76^*∗*^

^*∗*^Compared with group A, *P* < 0.05.

## Data Availability

The data used to support the findings of this study are available from the corresponding author upon request.
